# Use of Antipyrin with Quinine

**Published:** 1889-03

**Authors:** 


Use of Antipyrine with Quinine.—Derlon says (*Revue Gen. de Clin. et de Therap.*, July 15, 1888,) that if antipyrine is added to the prescription containing quinine, in cases in which large doses of quinine are necessary (as much as fifteen grains or more), the unpleasant effects of the quinine are obviated. He adds three grains of antipyrine to each five grains of quinine. This, he says, also increases the antipyretic effect of the quinine, and prevents quinism. The mixture is better tolerated by the stomach than the quinine alone. *Chicago Med. Jour. and Ex.*

---

A New Diagnostic Sign in Shoulder Presentation.—Quiz Master—"How do you diagnosticate a shoulder presentation?" Student—"By feeling for the hair in the axilla."—*Medical Record.*

